# Study on oxidation activity of Ce–Mn–K composite oxides on diesel soot

**DOI:** 10.1038/s41598-020-67335-5

**Published:** 2020-06-22

**Authors:** He Huang, Xiao Zhang, Junheng Liu, Song Ye

**Affiliations:** 1 School of Traffic Engineering, Nanjing Institute of Industry Technology, Nanjing, 210046 China; 2 Zhenjiang Campus, Army Military Transportation University of PLA, Zhenjiang, 212000 China; 3 School of Automotive and Traffic Engineering, Jiangsu University, Zhenjiang, 212013 China; 4 SAIC Volkswagen Automotive Company Limited, Shanghai, 201800 China

**Keywords:** Mechanical engineering, Carbon capture and storage

## Abstract

As an effective method, diesel particulate filter (DPF) technology has a great contribution in reducing soot emissions from diesel engines. To achieve passive regeneration of DPF at low temperatures, K-doped Ce_0.5_Mn_0.5_O_2_ catalysts were synthesized using sol–gel method. The effect of K-doped catalysts-K_z_–Ce_0.5_Mn_0.5_O_2_-on the oxidation of soot had been studied by thermogravimetric analysis, and the corresponding catalytic properties were evaluated based on X-ray diffraction (XRD), hydrogen temperature programmed reduction (H_2_-TPR), O_2_ temperature programmed desorption (O_2_-TPD) Raman spectroscopy (Raman), Brunauer–Emmett–Teller (BET) and Fourier-Transform-Infrared (FTIR).The results showed that K doping facilitated the oxidation of diesel particulate matter, which was indicated by the entire mass loss curve shifting to lower temperatures. K_0.2_–Ce_0.5_Mn_0.5_O_2_ showed the best performance among the series of K-doped catalysts. Compared with the findings for Ce_0.5_Mn_0.5_O_2_, the ignition temperature of soot oxidation (T_i_) had been lowered by 28 ℃, and the maximum peak combustion temperature (T_m_) of the dry soot decreased by 61 °C. Furthermore, compared with the Ce_0.5_Mn_0.5_O_2_-catalyzed reaction, K doping led to a lower activation energy and significantly improved pre-exponential factor. The minimum reaction activation energy of 27.46 kJ/mol was exhibited by K_0.2_–Ce_0.5_Mn_0.5_O_2_.

## Introduction

Diesel vehicles are currently attracting interest owing to their distinctive characteristics of superior power performance and fuel economic properties^1–3^ while the pollutants contained in diesel exhaust gas, particularly soot, pose hazards to human’s living environment and become one of the source of greenhouse gas which leads to global warming. Therefore, finding proper ways to cut down soot emissions from diesel engines become one of the researching hotpots in the study progress of diesel vehicles, that’ why many researchers studied diesel particulate filters (DPF), an effective methods of lowering soot emissions by passive regeneration by chemical catalysis and improving the temperature of the diesel exhaust which is sufficient for soot to burn out. Hence, selecting a suitable catalyst is the key challenge for regeneration^4–8^. It is well known that the utilization rate of rare earth resources is low, despite their abundance. CeO_2_ plays an important role in three-way catalysts owing to its excellent oxygen storage and release properties^9^. Generally, CeO_2_ performs as an oxygen buffer to store excess oxygen quickly as the oxygen concentration in the exhaust gas is increasing to higher level, and releasing oxygen promptly at low oxygen concentrations^10–12^. In addition, CeO_2_ is a promising noble metal substitute for improving the anti-poisoning performance and high temperature durability of catalysts^13,14^. The technology of Ce-based catalysts is still constantly improving after decades of development.

The unique chemical and physical properties of rare earth-based catalysts present due to their electronic energy level structure^15–17^. They are widely applied in numerous fields, such as automotive exhaust gas purification, fuel cells, and coatings. When applied for exhaust gas purification, rare earth-based catalysts enable simultaneous conversion of hydrocarbons (HCs), CO, and NO_x_ in the exhaust gas into H_2_O, CO_2_, and N_2_^18,19^. MnO_x_ is a strong oxidizing agent, therefore when it forms a solid solution with Ce the mobility of oxygen species is effectively promoted by the significantly increased number of oxygen vacancies, and the catalytic oxidation activity is significantly improved^20^.

The application of CeO_2_ in diesel catalysis has been one of the hotpots of intensive studies in recent centuries, with most studies focusing on elemental doping of CeO_2_^21–23^. CeO_2_-based composite oxides obtained by doping with Fe, Zr, La, Pr, and Nd show stronger catalytic activity than CeO_2_ alone^24,25^. Kohn et al.^26^ doped CeO_2_ with Sm and La via coprecipitation, and the doped CeO_2_ material was found to display a smaller subgrain size and larger specific surface area. By doping CeO_2_ with Mn and Cu, March et al.^27^ found that Mn ions entered the CeO_2_ lattice to form a solid solution, which exhibited a larger number of oxygen vacancies and a significantly increased concentration of surface adsorbed oxygen. The Cu ions were found to disperse on the surface of the CeO_2_ fluorite structure, and the interaction between Cu and Ce enabled rapid release of lattice oxygen in a reducing atmosphere. The formation of the Ce–Mn solid solution promotes the mobility of oxygen species, and leads to a significantly increased number of oxygen vacancies. Studies have shown that the catalyst maintains the basic cubic fluorite structure of CeO_2_ when the Ce/Mn ratio is greater than 1^28^. To address the high-temperature sintering of CeO_2_, Hemeryck et al.^29^ doped Ce–Mn catalyst with Ba, which prevented the separation of the Ce–Mn phases and was found to be effective for suppressing the sintering of oxides.

Our previous studies show that Mn enters the crystal lattice of CeO_2_ to form a Ce–Mn solid solution^30^. The introduction of Mn induces valence state variation of Ce^4+^/Ce^3+^, and increases the number of surface oxygen vacancies. The synergistic effect of Ce and Mn enhances the catalyst selectivity for soot oxidation, leading to significantly enhanced catalytic activity. Ce_0.5_Mn_0.5_O_2_ was selected as the base material as it displayed the highest activity in our previous research. To further lower the soot oxidation temperature, K was doped into Ce_0.5_Mn_0.5_O_2_, which was found to exhibit the highest activity in our previous research. The sol–gel method was used to synthesize a series of K_z_–Ce_0.5_Mn_0.5_O_2_ catalysts. To determine the optimal K doping ratio, the K-doped catalysts were characterized and their catalytic activities were evaluated. The findings revealed the influence of K doping on the crystal structure of Ce–Mn catalyst and the oxygen species mobility, as well as the consequent valence variation of the Ce and Mn ions. Furthermore, the effect of K doping on diesel particulate matter oxidation was analyzed, providing reference for future studies on the catalyzed reaction of diesel particulate matter and catalyst coating of DPF.

## Experimental

### Catalyst preparation

The group of K_z_–Ce_0.5_Mn_0.5_O_2_ catalysts was synthesized using the sol–gel method. For a typical synthesis, Mn(NO_3_)_2_ and Ce(NO_3_)_3_·6H_2_O with a particular stoichiometric ratio were thoroughly mixed in deionized water and then KNO_3_ was introduced into the mixture. The amount of KNO_3_ required was calculated according to the ratio of K in the final product. After the solution was magnetically stirred at a constant temperature for 5 min, citric acid of the equivalent mole amount to that of metal cations in the solution was added. The mixed solution was then ultrasonicated for 10 min, before being placed in a water bath at a constant temperature of 80 °C. The mixed solution was magnetically stirred in the water bath until it formed a gel. The newly-formed gel was dried overnight at 120 °C in a blast dryer and a muffle furnace was utilized to calcined the dried product for 4 h. The catalysts synthesized were denoted K_*z*_–Ce_0.5_Mn_0.5_O_2_ (z = 0.1, 0.2, 0.3), with the specific formulas being K_0.1_–Ce_0.5_Mn_0.5_O_2_, K_0.2_–Ce_0.5_Mn_0.5_O_2_, and K_0.3_–Ce_0.5_Mn_0.5_O_2_, separately.

### Catalyst characterization method

The crystal structure of the catalyst samples was analyzed by the Burker D8 Avdance X-ray diffraction (XRD). The instrumental test conditions were as follows: running at specific condition (40 kV, 30 mA), utilizing Cu Kα radiation (λ = 0.15418 nm) filtered with nickel. The scanning range of small angle diffraction was 2 = 0.8°–8° and the scanning step length was 0.002°. The scanning range of wide angle diffraction was 2 = 10°–80° and the scanning step length was 0.02°.The scanning step is 4°/min.

H_2_-temperature programming reduction (H_2_-TPR) was performed on a Chemisorb 2720 pulse chemisorption system equipped with a TPx (temperature-programmed controller and software) system and a TCD detector (Micromeritics). 10 mg of the sample was heated up to 700 ℃ from room temperature with a heating addition rate of 10 ℃/min. The reducing atmosphere gas (the mixture of 10 vol% H_2_ and N_2_) was supplied at a flow rate of 25 mL/min^31^.

The TPDRO 1100 instrument was used for the O_2_ temperature-programmed desorption (O_2_-TPD) O_2_-TPD experiment. The specific experimental steps are as follows: 200 mg of catalyst was weighed into the sample cell; then, the temperature was raised to 600 °C at a heating rate of 15 °C/min in an O_2_ atmosphere of 20 mL/min, and the temperature was maintained for 30 min. Finally, after the temperature drops to room temperature, oxygen desorption rises 900 °C at 10 °C/min in He atmosphere from room temperature. Same conditions were applied in these O_2_-TPD experiments to control variable and maintain accuracy. A TCD detector was used to characterize and observe the concentration signals of the desorbed O_2_.

The Raman spectrometer used in the experiment was a Renishaw micro Raman spectrometer produced by Renishaw, Germany. It uses a CCD multi-channel detector, an excitation light source is a 633 nm He–Ne ion laser, and the resolution of the Raman dynamic line is 0.5/cm.

The N_2_ adsorption–desorption isotherm was determined on the ASAP2020 physical adsorption apparatus. Before the test, the catalyst was fully ground in a mortar, about 0.1 g of the sample was weighed, and a pre-degassing treatment was performed under a vacuum condition at 200 °C for 10 h. Then, the liquid nitrogen was used as the adsorption medium, the adsorption and desorption volumes of the catalyst for N_2_ at different pressures (relative pressure range 0.0–1.0) were tested at 77 K to obtain the adsorption–desorption isotherms of the samples.

The Fourier-Transform-Infrared (FTIR) spectrum of the sample was recorded on a Bruker Tensor 27 spectrometer. The instrument resolution was 4/cm and 32 scans were accumulated. The in situ pool without sample was scanned in He atmosphere to obtain the background spectrum. The composite catalyst with a load of K adsorbed carbon smoke was pressed into self-supporting sheets with a thickness of 7.5 mg/cm^2^, which were fixed in the in-situ infrared transmission pool, which was connected to the air path system and heated to 500 ℃. After the sample was pretreated at 200 ℃ for 1 h and reduced to room temperature, 100 mL/min of high purity He gas was injected, and the temperature was raised to 500 ℃ at 5 ℃/min, and the temperature was kept at 500 ℃ for 3 h, during which the FTIR spectrum was recorded.

### Catalytic testing

T_SOF_, T_pre_, T_m_, and T_i_ were used to evaluate the catalyst activity in this paper.They indicate the temperatures corresponding to the mass loss peaks of the soluble organic fraction (SOF), soot precursor, and dry soot, and the soot ignition temperature (the temperature corresponding to 10% soot mass loss), respectively.

In Table 1, the main technical parameters of the test diesel engine were listed. TGA/DSC1 thermogravimetric analyzer from Swiss METTLER company was used for the thermogravimetric analysis (TGA) of particle samples, with a high precision microgram electronic balance and temperature sensor. The PM samples data was collected by AVL SPC472 partial flow point collection system. An oxygen atmosphere with a mass fraction of 12% was selected for thermogravimetric test, which was close to the oxygen content in diesel exhaust. N_2_ in high-purity had been performed as the protective gas, and its flow velocity was at a constant rate of 100 mL/min under the setting temperature from 40 to 800 ℃, and the heating addition rate was maintained at 15 ℃/min. The sample weight was 3 mg. Mixed at the ratio of 4:1 evenly, the tested diesel soot particles and catalyst became an uniform mixture.

**Table 1:** Specifications of testing engine. The methods described below have been reproduced in part from^32^.

Item	Specification
Type	4-cylinder, in-line, turbocharged and intercooled
Bore × stroke (mm)	105 × 118
Combustion chamber type	Direct injection ω type
Compression ratio	17.5
Displacement (L)	4.09
Max./torque/speed (Nm/r/min)	400/1,500
Rated power/speed (kW/r/min)	95/2,600
Max. injection pressure (MPa)	160
Fuel injection system	Electronic controlled high pressure common-rail

## Results and discussion

### Activity tests

**Figure 1:**
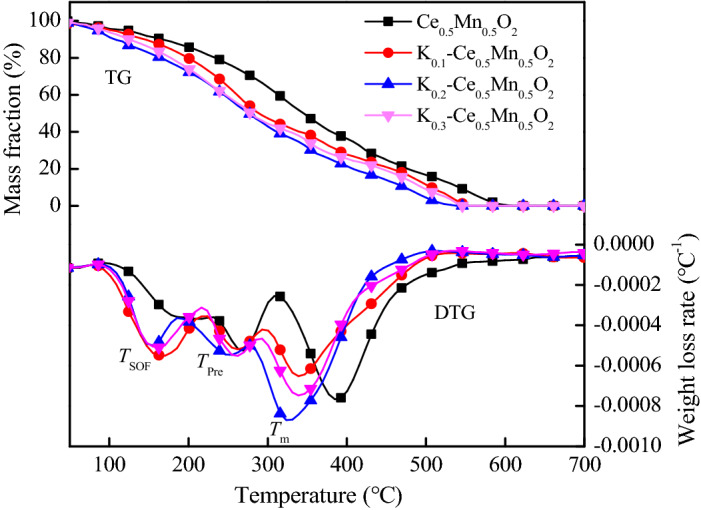
TG and DTG curves of diesel particulate matter with catalysts.

The thermogravimetric (TG) and differential thermogravimetric (DTG) curves of diesel particulate matter with the catalysts are presented in Fig. 1. It is obvious that the mass loss rate peaks of the soluble organic fraction (SOF), soot precursor, and dry soot, shifted significantly to lower temperatures with the doping of K. The introduction of K significantly lowered the oxidation temperature of diesel exhaust particulates compared with the catalytic activity of Ce_0.5_Mn_0.5_O_2_. The catalyst K_0.2_–Ce_0.5_Mn_0.5_O_2_ (z = 0.2) displayed the best catalytic effect on diesel soot oxidation.

The weight loss characteristics of diesel particulate matter in the presence of various catalysts are shown in Table 2. It is noticeable in the table that K doping reduced the oxidation temperature of SOF. Specifically, with increasing K content, the SOF oxidation temperature decreased by 11, 19, and 18 °C, respectively. This indicates that the ability of the Ce–Mn solid solution to oxidize diesel particulate matter at low temperatures was improved by K doping. In the intermediate temperature range, the oxidation temperature of the soot precursor decreased by 15, 25, and 23 °C compared with the Ce_0.5_Mn_0.5_O_2_-catalyzed reaction, with K_0.1_–Ce_0.5_Mn_0.5_O_2_, K_0.2_–Ce_0.5_Mn_0.5_O_2_, and K_0.3_–Ce_0.5_Mn_0.5_O_2_ as the catalyst, respectively. As indicated by the data, K_0.2_–Ce_0.5_Mn_0.5_O_2_ displayed better relative activity for the catalytic oxidation of soot precursor. A similar trend was also observed in the high temperature range. K_0.1_–Ce_0.5_Mn_0.5_O_2_, K_0.2_–Ce_0.5_Mn_0.5_O_2_, and K_0.3_–Ce_0.5_Mn_0.5_O_2_ reduced the ignition temperature of dry soot by 17, 28, and 26 °C, respectively, compared with the value obtained using Ce_0.5_Mn_0.5_O_2_. Meanwhile, the maximum peak combustion temperature of dry soot was also lowered by 45, 61, and 57 °C, upon K doping (*z* = 0.1, 0.2, and 0.3, respectively). Therefore, K doping significantly enhanced the catalytic activity of the Ce–Mn solid solution for the oxidation of particulate matter, and lowered the temperature required for soot oxidation.

**Table 2:** Weight loss characteristics of diesel particulate matter.

Catalyst	*T*_SOF_ (°C)	*T*_pre_ (°C)	*T*_i_ (°C)	*T*_m_ (°C)
Ce_0.5_Mn_0.5_O_2_	172	274	306	384
K_0.1_–Ce_0.5_Mn_0.5_O_2_	161	259	289	339
K_0.2_–Ce_0.5_Mn_0.5_O_2_	153	249	278	323
K_0.3_–Ce_0.5_Mn_0.5_O_2_	154	251	280	327

**Figure 2:**
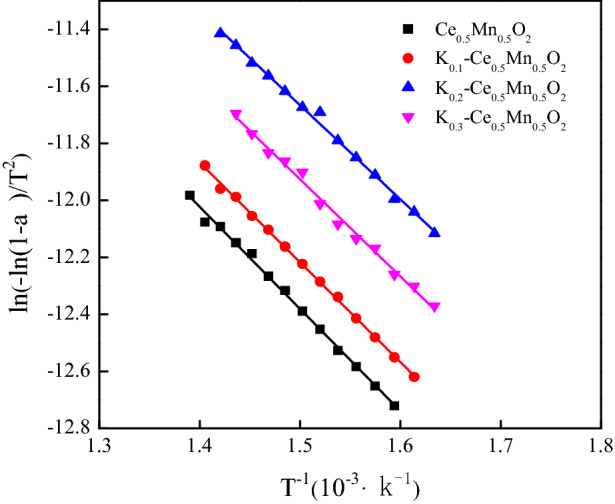
Fitting curves of ln[− ln(1 − α)/T^2^] and of 1/T particles under catalysis.

The Coats–Redfern integral was determined for the catalyzed diesel particulates, and the linear fitting curves of ln[− ln(1 − α)/T^2^] versus 1/T are shown in Fig. 2. The fitting curves showed excellent linear regression, with the goodness of fit (R^2^) exceeding 0.99, indicating high accuracy of the fitting results. The activation energy and pre-exponential factor of each reactant can be obtained by calculating the reaction curve equation of each reactant with Ce_0.5_Mn_0.5_O_2_ and K_z_–Ce_0.5_Mn_0.5_O_2_. The calculation results are listed in Table 3. Doping with K led to a decreasing trend in reaction activation energy, implying that the energy required for catalytic oxidation of soot was lower with K-doped catalyst, which resulted in easier soot oxidation. The minimum observed activation energy of 27.46 kJ/mol was achieved for the reaction catalyzed by K_0.2_–Ce_0.5_Mn_0.5_O_2_, which is about 20 kJ/mol lower than that reported in relevant literature^33,34^. Moreover, the pre-exponential factor of soot oxidation was found to increase significantly with K-doped Ce_0.5_Mn_0.5_O_2_ as the catalyst. A larger pre-exponential factor represents more effective collisions between the catalyst and soot during the reaction process, which would facilitate soot oxidation.

**Table 3:** Effect of K_z_–Ce_0.5_Mn_0.5_O_2_ catalyst on activation energy and pre-exponential factor.

Samples	Fitting curve equation	Activation energy (kJ/mol)	Preexponential factor (/min)
Ce_0.5_Mn_0.5_O_2_	y = − 3.58x − 7.01	29.77	48.48
K_0.1_–Ce_0.5_Mn_0.5_O_2_	y = − 3.50x − 6.97	29.10	49.49
K_0.2_–Ce_0.5_Mn_0.5_O_2_	y = − 3.30x − 6.71	27.46	60.23
K_0.3_–Ce_0.5_Mn_0.5_O_2_	y = − 3.41x − 6.81	28.34	56.16

### Catalyst characterization

**Figure 3:**
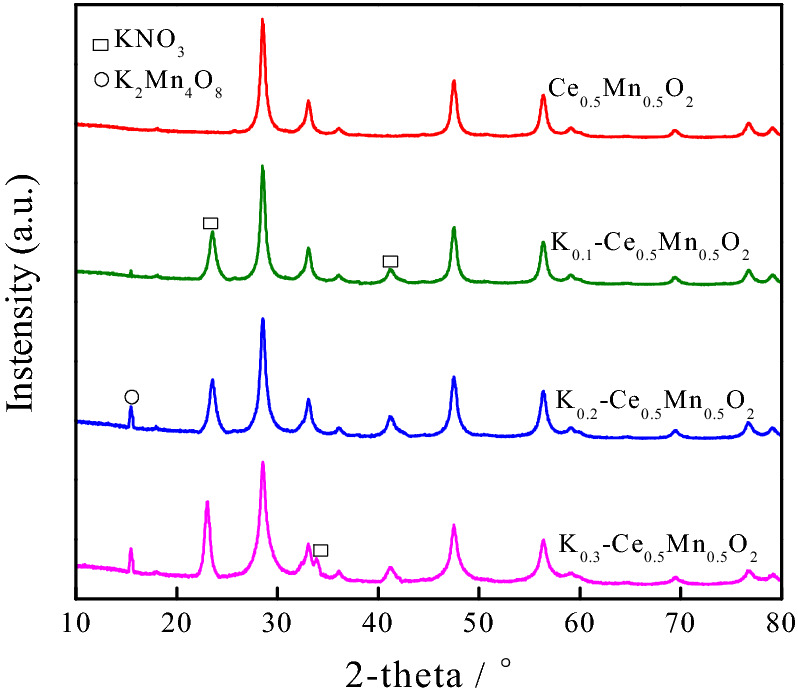
XRD profiles of Ce_0.5_Mn_0.5_O_2_ and K_*z*_–Ce_0.5_Mn_0.5_O_2_ catalysts.

Some typical characterizations had been taken to investigate the mechanism of soot catalytic oxidation by K_z_–Ce_0.5_Mn_0.5_O_2_ catalysts on the microstructure level. The XRD profiles of Ce_0.5_Mn_0.5_O_2_ and K_*z*_–Ce_0.5_Mn_0.5_O_2_ (z = 0.1, 0.2, and 0.3) are shown in Fig. 3. Compared with the standard XRD profile of pure CeO_2_ (JCPD 34-0394), the K-doped catalysts exhibited a typical fluorite structure, with the diffraction peaks of CeO_2_ locating at 28.5°, 33.1°, 47.5°, 56.3°, 59.0°, 69.6°, and 76.9°. The fluorite structure of the material was therefore not altered by K doping. However, the diffraction peaks of the K-doped catalysts shifted to lower angles. This is because the antifluorite structure of K_2_O enabled the formation of coordinating tetrahedra between K and Ce, with K partially entering Ce_0.5_Mn_0.5_O_2_ to form a solid solution. Since the ion radius of K^+^ (0.133 nm) is larger than that of Ce^4+^ (0.094 nm), Ce^3+^ (0.103 nm), Mn^3+^ (0.065 nm), and Mn^4+^ (0.053 nm), the K ions doped into the lattice induced lattice expansion and enlarged the unit cells in the fluorite structure, leading to the diffraction peaks shifting. In addition, Ce^4+^ was partially substituted by K^+^ during the doping process, which was accompanied by the transition of electrons between ions, generating oxygen vacancies.

Figure 3 also indicates that upon K doping, the characteristic peaks of KNO_3_ appeared at 2θ = 23.5° and 41.8°. With the continuous increase of K content, a peak at 2θ = 33.8°, which is also attributed to KNO_3_, was observed for K_0.3_–Ce_0.5_Mn_0.5_O_2_. Meanwhile, the characteristic diffraction peak at 2θ = 15.5° of a new compound K_2_Mn_4_O_8_ also appeared for catalysts with higher K content. The K_2_Mn_4_O_8_ phase was absent and the peak positions of the Mn oxide phase were shifted to lower angles for the catalyst with low K content (K_0.1_–Ce_0.5_Mn_0.5_O_2_). The formation of K_2_Mn_4_O_8_ at elevated K content is attributed to the fact that in addition to forming the tetrahedral structure with Ce, K also combines with Mn oxides and dissolves in the solid solution. Therefore, at higher K concentrations, in addition to the K species covering the surface of the solid solution, residual K combined with the Mn oxide to form a new K_2_Mn_4_O_8_ phase.

**Figure 4:**
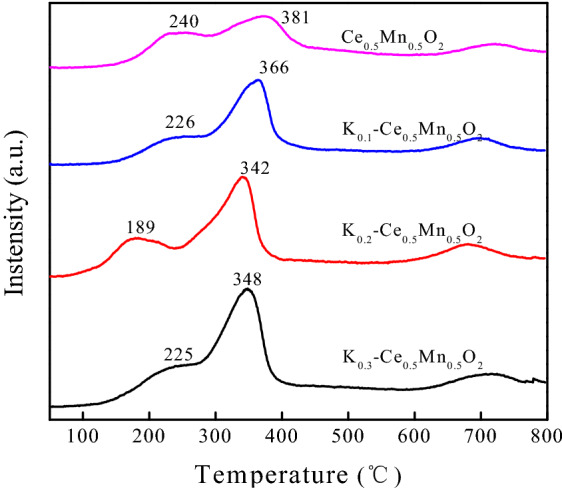
H_2_-TPR curves of K_z_–Ce_0.5_Mn_0.5_O_2_ catalysts.

The redox capacity is an important indicator of the catalytic performance of a catalyst, particularly in cases where the catalyst is applied for catalytic oxidation of diesel particulate matter. H_2_-TPR is an effective technique that reflects the reducing ability of a catalyst. Comparing the H_2_-TPR profiles of Ce_0.5_Mn_0.5_O_2_ and K_*z*_–Ce_0.5_Mn_0.5_O_2_ (Fig. 4) revealed that the catalytic peak positions varied depending on the amount of K doped. The reactive oxygen species present can be characterized using the temperatures corresponding to the reduction peaks, and the amount of reactive oxygen species represents the catalytic ability of the catalyst in the oxidation of particulate matter. In our previous work, Ce_0.5_Mn_0.5_O_2_ displayed relatively good reduction peak positions in the range of 100–500 °C, indicating high activity, which was a result of the conversion and electron transition between Mn^4+^/Mn^3+^ and Ce^4+^/Ce^3+^ ion pairs^32^. The peak positions of the H_2_-TPR profile varied with increasing K content (Fig. 4). Compared with the peak positions of Ce_0.5_Mn_0.5_O_2_ at 240 and 381 °C, the corresponding peak temperatures of K_0.1_–Ce_0.5_Mn_0.5_O_2_ dropped to 226 and 366 °C, respectively. Therefore, K doping resulted in the shift of the reduction peaks (< 400 °C) to lower temperatures.

The K^+^ ions doped into the material substituted some of the Ce^4+^ ions, which caused greater conversion of Mn^3+^ to the higher valence state (Mn^4+^), leading to a gradually increasing Mn^4+^/Mn^3+^ ratio with higher K^+^ content. Consequently, the surface charge of the catalyst became unbalanced, and the mobility of lattice oxygen was enhanced. The generation of oxygen vacancies then enhanced the adsorption of reactive oxygen species by the catalyst^35^. The H_2_ reduction peaks indicated that the K^+^ ions remained relatively stable without going through valence state conversion. Higher degrees of K doping led to a mounting in the amount of oxygen species adsorbed by the catalyst and significantly improved oxygen mobility. It is speculated that for the K-doped catalysts the active oxygen species on K sites could spill over to the soot surface, and react with the free carbon sites to form ketene species with C=C=O structure; the active oxygen species at K sites were then continuously supplied with gaseous oxygen via mobile lattice oxygen until the particulate matter was fully oxidized.

**Figure 5:**
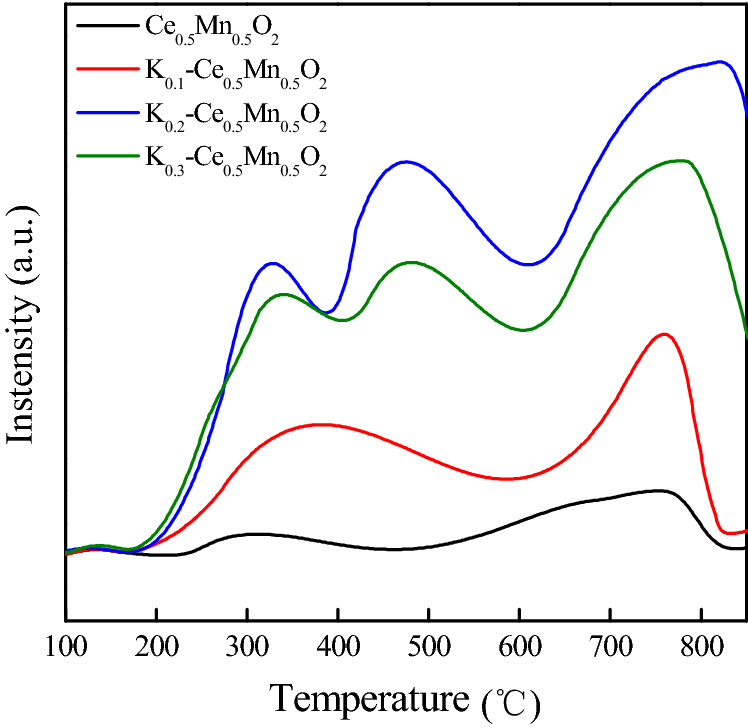
O_2_-TPD profiles of Ce_0.5_Mn_0.5_O_2_ and K_*z*_–Ce_0.5_Mn_0.5_O_2_ catalysts.

O_2_-TPD is a technique that indicates the activity of a catalyst and its selectivity for reactant molecules. Generally, there are three typical kinds of oxygen species on the surface of Ce-based composite catalysts, including adsorbed molecular oxygen (O_2_^−^) that desorbs in the low temperature region, adsorbed atomic oxygen (O^−^) that desorbs in the intermediate temperature region, and lattice oxygen (O^2−^) that desorbs in the high temperature region. The O_2_-TPD profiles of Ce_0.5_Mn_0.5_O_2_ and K_*z*_–Ce_0.5_Mn_0.5_O_2_ catalysts are presented in Fig. 5. The observation of weak low-temperature desorption taken for all of the catalysts, indicating a low proportion of adsorbed molecular oxygen (O_2_^−^). K doping diversified the flow mode of oxygen species, and supplemented the cubic fluorite structure defects caused by Mn entering the CeO_2_ lattice, which facilitated the migration of oxygen species, the conversion of lattice oxygen to adsorbed oxygen, and the adsorption of surrounding oxygen molecules.

As shown in Fig. 5, K_0.1_–Ce_0.5_Mn_0.5_O_2_ (K content of z = 0.1) showed a significantly higher desorption peak intensity in the intermediate temperature region, indicating enhanced oxygen species activity and an increased amount of adsorbed atomic oxygen (O^−^). The adsorbed atomic oxygen (O^−^) performs a dominant part in the catalytic oxidation of diesel soot. However, the corresponding desorption temperature in the intermediate temperature region increased for K_0.1_–Ce_0.5_Mn_0.5_O_2_ compared with Ce_0.5_Mn_0.5_O_2_. This is thought to be caused by the improved stability among atoms induced by the small amount of K during the calcination of the catalyst, which inhibited the desorption capability of the adsorbed oxygen species. The oxygen desorption intensity was significantly enhanced with further increase of the K content. When z = 0.2, the adsorbed atomic oxygen (O^−^) actively desorbed, displaying a distinct oxygen desorption peak at ~ 480 °C. In addition, the lattice oxygen desorption at 700 °C was also intensified.

**Figure 6:**
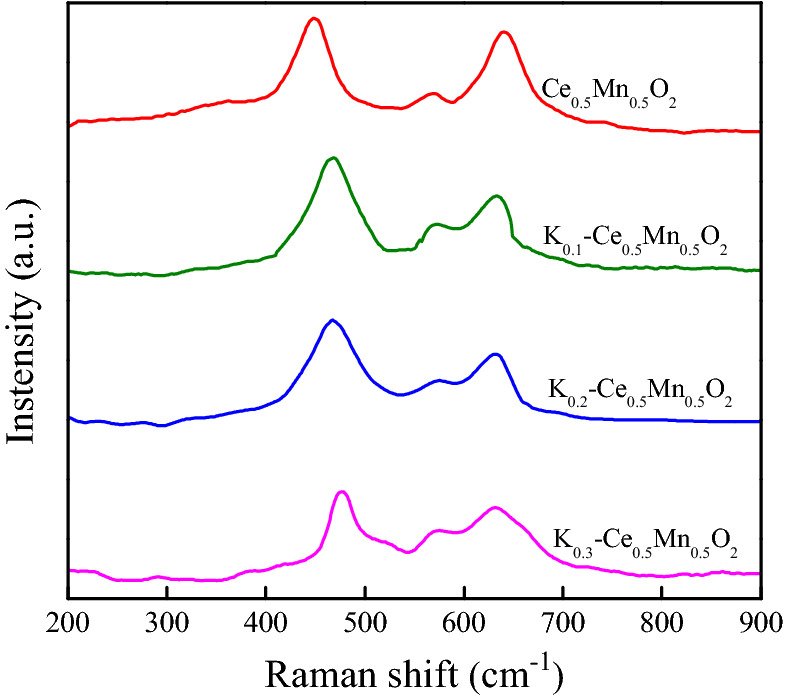
Raman spectra of Ce_0.5_Mn_0.5_O_2_ and K_z_–Ce_0.5_Mn_0.5_O_2_ catalysts.

The Raman spectra of Ce_0.5_Mn_0.5_O_2_ and K_*z*_–Ce_0.5_Mn_0.5_O_2_ catalysts are shown in Fig. 6. Ce_0.5_Mn_0.5_O_2_ exhibited a typical cubic fluorite structure, indicated by a vibration peak (447/cm), which was attributed to the typical F_2g_ vibration of the CeO_2_ cubic fluorite structure. The peak shifted slightly upon K doping, however, the overall cubic fluorite structure of CeO_2_ was not altered, which is accordant with the XRD results. In our previous work we reported that the vibration peak at 641/cm was caused by Mn entering the CeO_2_ lattice. Characteristic peaks at the same position were also observed for K_*z*_–Ce_0.5_Mn_0.5_O_2_ catalysts, which were attributed to the vibration of Mn–O. However, the peaks were broader and showed a slight red shift. This observation suggests the existence of a small amount of MnO_x_ in the K_*z*_–Ce_0.5_Mn_0.5_O_2_ catalysts, and that K doping led to the variation of the structural valence between Mn and Ce, which subsequently resulted in a change in the amount of oxygen vacancies, facilitating the migration of oxygen species on the catalyst surface and promoting the catalytic combustion of soot.

Table 4 exhibits the information of the three CeO_2_ samples, which are surface area, pore volume and average pore diameter. The specific surface area of the sample was calculated using BET (Brunauer–Emmett–Teller) method, and the pore volume and aperture were calculated by the isotherm adsorption branch using BJH (Barrett–Joyner–Halenda) model, where the pore volume was calculated using the adsorption volume at the relative pressure p/p0 = 0.99. It can be seen from Table 4 that the surface area of the prepared K_z_–Ce_0.5_Mn_0.5_O_2_ is significantly greater than that of Ce_0.5_Mn_0.5_O_2_. The larger surface area indicates more surface active sites per unit mass of K_*z*_–Ce_0.5_Mn_0.5_O_2_. Furthermore, there are more opportunities to achieve a closer contact between the catalysts and the reactants.

**Table 4:** Surface area, pore volume and pore diameter of CeO_2_ samples.

Sample	Surface (m^2^/g)	Pore volume (cm^3^/g)	Average pore diameter (nm)
Ce_0.5_Mn_0.5_O_2_	52	0.19	6.2
K_0.1_–Ce_0.5_Mn_0.5_O_2_	68	0.17	5.7
K_0.2_–Ce_0.5_Mn_0.5_O_2_	89	0.14	5.4
K_0.3_–Ce_0.5_Mn_0.5_O_2_	70	0.16	5.6

**Figure 7:**
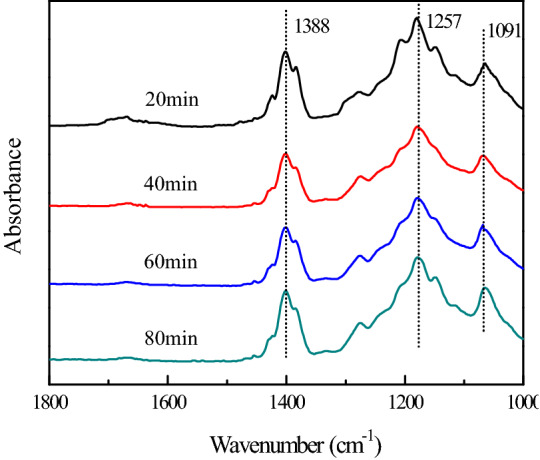
In situ FTIR spectra of the mixture of soot and K_0.2_–Ce_0.5_Mn_0.5_O_2_ after heating at 500 °C.

In situ FTIR results are shown in Fig. 7. It is found that ketene species may exist in this reaction (1388/cm). At first, the mixture of soot and K_0.2_–Ce_0.5_Mn_0.5_O_2_ was heated to 430 °C in O_2_ + He followed by cooling down to 200 °C with purging with He. In this step, some soot was depleted and thus a clear FTIR signal and lots of free carbon sites were obtained. The corresponding spectra are shown in Fig. 7 (20 min), illustrating the presence of chelating bidentate carbonate and ionic carbonate on K_0.2_–Ce_0.5_Mn_0.5_O_2_. Because diesel engine exhaust contains a large amount of NO, the real exhaust atmosphere is simulated. NO was introduced and switched off when the spectrum did not change significantly. As expected, the band of the ionic nitrite was observed in Fig. 7 (40 min). At this time, free carbon sites and ionic nitrite were abundant on the surfaces of soot and K_0.2_–Ce_0.5_Mn_0.5_O_2_, respectively.

The mixture was progressively heated up to higher temperatures in He (60 and 80 min). During this period, the band of the ionic nitrite gradually decreases in intensity, simultaneously with the formation of the ketene group. These facts suggest that the ionic nitrite may be consumed with the production of the ketene group. In other words, the ionic nitrite on K_0.2_–Ce_0.5_Mn_0.5_O_2_ interacts with the free carbon sites on the soot to form the ketene group, which can be described as^36^:

1 $$ {\text{C}} = {\text{C}}^{*} + {\text{K}}^{ + } - {\text{NO}}_{{2}}^{ - } \to {\text{C}} = {\text{C}} = {\text{O}} + {\text{NO}} + {\text{K}}^{ + } -$$

The ketene group has been identified as the intermediate of soot oxidation with O_2_ or NO_2_, which is a surface oxygen complex formed on the surface of soot with graphite structures. Likewise, the ketene group can serve as the intermediate of soot oxidation with NO. During this process, chelating bidentate carbonate (1,257/cm) and ionic carbonate (1,091/cm) are formed, which have been observed in soot oxidation with O_2_. These carbonates originate from the adsorption of the produced CO_2_ on potassium sites.

### Catalytic characteristics

**Figure 8:**
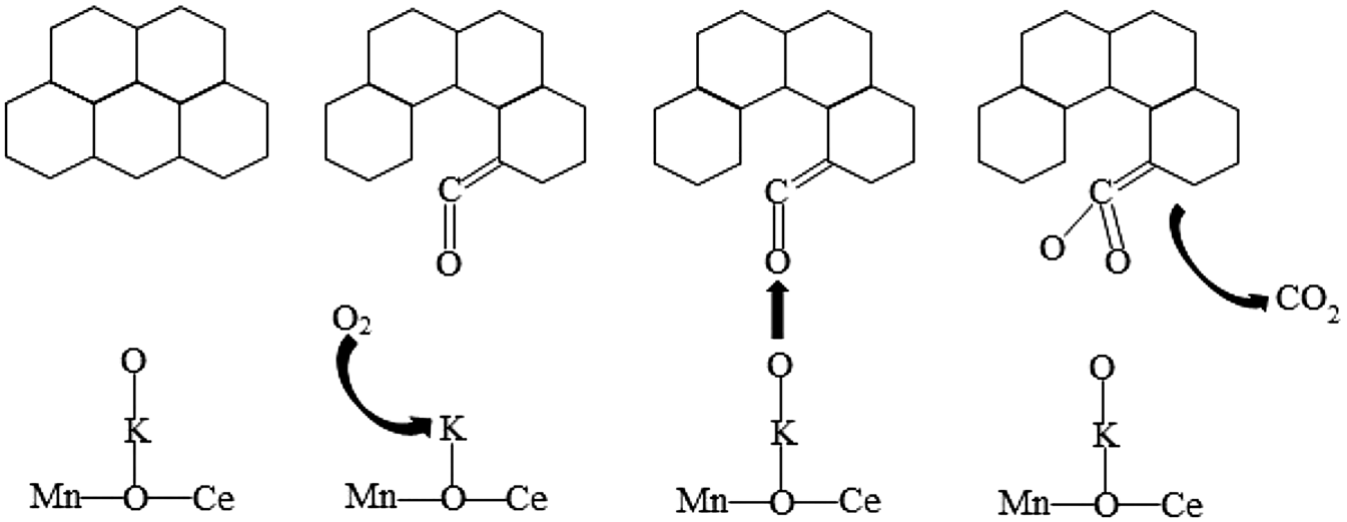
Illustration of particle matter combustion with O_2_ on K_*z*_–Ce_0.5_Mn_0.5_O_2_.

The catalytic oxidation of soot using K-doped Ce_0.5_Mn_0.5_O_2_ as the catalyst was analyzed by relating the characterization results indicating variations in structure and surface ions, to the redox capacity of K-doped Ce_0.5_Mn_0.5_O_2_, as shown in Fig. 8. For the K-doped Ce_0.5_Mn_0.5_O_2_ catalysts, the active oxygen species on the K sites spill over to the soot surface and react with the free carbon sites to form ketene species with C=C=O structure. Meanwhile, the K sites enable the supplementation of active oxygen species in the catalyst by activating surrounding gaseous oxygen and enhancing the mobility of lattice oxygen until the soot is fully catalytically oxidized to CO_2_^37^. The activated oxygen on the surface of K site may overflow to the free carbon site on the soot, forming a carbon–oxygen complex, that is reaction intermediate, ketene group. The K effect is used to supplement the consumed surface oxygen by chemical adsorption and dissociation of gas-phase oxygen or surface lattice oxygen. In the absence of transient reactions, carbothermal reduction and gas phase oxygen, surface lattice oxygen participates in soot combustion. The ketene group is further oxidized to carbon dioxide by other active oxygen, which increases the number of exposed free carbon sites. The selectivity of soot combustion is due to the fact that these free carbon can be directly oxidized into CO by gas phase oxygen. The number of active sites increases with the increasing of K, which will occupy more free carbon sites and avoid combining with gas phase O_2_ to form CO, resulting in a small increase in CO_2_ selectivity. What’s more, K can promote the escape of oxygen to soot by forming ketene group.

## Conclusions

In the current study a series of K-doped Ce_0.5_Mn_0.5_O_2_ catalysts were synthesized using the citrate sol–gel method. Their ability to catalyze diesel particulate matter oxidation was investigated by thermogravimetric analysis. The activation energy and pre-exponential factor of each reaction were determined by further analysis of particle oxidation kinetics. The catalytic mechanism was explored on the microstructure level by means of XRD, H_2_-TPR, O_2_-TPD, and Raman. The following conclusions were reached:

1. K doping promoted the oxidation of diesel particulate matter, which manifested as the entire mass loss curve shifting to lower temperatures. Compared with the Ce_0.5_Mn_0.5_O_2_-catalyzed reaction, the ignition temperature of soot decreased by 17, 28, and 26 °C, with K_*z*_–Ce_0.5_Mn_0.5_O_2_ (z = 0.1, 0.2, 0.3, respectively) as the catalyst. While, the maximum peak combustion temperature of dry soot was also lowered by 45, 61, and 57 °C, upon K doping (*z* = 0.1, 0.2, and 0.3, respectively). The prepared K_*z*_–Ce_0.5_Mn_0.5_O_2_ has a larger specific surface area and the catalytic activity of K_*z*_–Ce_0.5_Mn_0.5_O_2_ increases with its specific surface area increasing. Doping with K significantly enhanced the catalytic activity of the Ce–Mn solid solution for special matter oxidation and reduced the oxidation temperature of soot.
2. Analysis of the oxidation kinetics of diesel particulates indicated lower activation energies and increased pre-exponential factors for reactions catalyzed by K-doped Ce_0.5_Mn_0.5_O_2_. The minimum observed activation energy of 27.46 kJ/mol was achieved using K_0.2_–Ce_0.5_Mn_0.5_O_2._ The oxygen species on K sites played an important role in soot oxidation, and were continuously supplemented by activating surrounding oxygen molecules to complete the oxidation process.
3. The K_*z*_–Ce_0.5_Mn_0.5_O_2_ catalysts synthesized using the citrate sol–gel method displayed the original cubic fluorite structure of CeO_2_. Therefore, material structures remained stable upon K doping. However, the valence states of Ce and Mn ions were altered by K doping. The amount of oxygen vacancies on the catalyst surface increased when the flow mode of the oxygen species diversified.
4. K doping also resulted in more adsorbed oxygen species and significantly improved oxygen mobility. In addition, the structural balance between Mn and Ce was also altered by K doping, which resulted in changes of the amount of oxygen vacancies, facilitating the migration of oxygen species on the catalyst surface and promoting the catalytic combustion of soot.

## Data Availability

All data included in this study were obtained by contacting the corresponding authors.
